# Long-read sequencing of clinical multidrug-resistant *Mycobacterium tuberculosis* reveals a novel *ethA* structural variant conferring ethionamide resistance

**DOI:** 10.3389/fmicb.2026.1929307

**Published:** 2026-10-27

**Authors:** Qiongdan Wang, Yaya Geng, Lianpeng Wu, Dandan Xia, Ruini Li, Baozhu Hu, Shuai Gao, Jinfang Lu, Jianyi Dai, Feng Xu, Xiaoqun Zheng, Guangxin Xiang

**Affiliations:** 1 Department of Laboratory Medicine, The First Affiliated Hospital of Wenzhou Medical University, Wenzhou, Zhejiang, China; 2 School of Laboratory Medicine and Life Sciences, Wenzhou Medical University, Wenzhou, Zhejiang, China; 3 Department of Clinical Laboratory, Wenzhou Central Hospital, Wenzhou, Zhejiang, China; 4 Department of Infectious Diseases, Wenzhou Central Hospital, Wenzhou, Zhejiang, China; 5 Key Laboratory of Laboratory Medicine, Ministry of Education, Wenzhou, Zhejiang, China

**Keywords:** ethionamide resistance, long-read sequencing, multidrug resistance, *Mycobacterium tuberculosis*, structural variation

## Abstract

Multidrug-resistant *Mycobacterium tuberculosis* (MDR-MTB) poses a major obstacle to global tuberculosis control. Although whole-genome sequencing (WGS) has advanced the detection of resistance-associated mutations, the contribution of structural variants (SVs) remains inadequately characterized due to the inherent limitations of short-read sequencing technologies in resolving repetitive genomic regions and large insertions or deletions. We applied Oxford Nanopore long-read sequencing to clinical MDR-MTB isolates and susceptible controls, complemented by Illumina short-read sequencing. Variants were identified using BCFtools and independently validated with SVIM-asm. Candidate resistance mutations were mapped against the 2023 World Health Organization (WHO) mutation catalogue. Functional validation of identified SVs was performed through heterologous expression in *Mycobacterium smegmatis*, minimum inhibitory concentration determination, Western blotting, and AlphaFold 3-based structural modeling. We identified two unclassified SVs within WHO-catalogued resistance genes: an *IS6110* insertion in *Rv0678* (c.59_60ins1358bp, p.Gln22fs) and a 131-bp deletion in *ethA* (c.135_265del, p.Trp45fs). While *IS6110*-mediated disruption of *Rv0678* has been previously associated with bedaquiline resistance, the *ethA* deletion represents a previously undescribed SV. Heterologous expression of the *ethA* deletion in *Mycobacterium smegmatis* conferred a 16-fold increase in the minimum inhibitory concentration of ethionamide (256 μg/mL vs. 16 μg/mL for wild-type), and AlphaFold 3 structural prediction provided a structural rationale for the loss of the C-terminal domain essential for monooxygenase activity. This study identifies a novel *ethA* SV conferring ethionamide resistance and provides functional validation of a specific *Rv0678 IS6110* insertion allele, demonstrating the power of long-read sequencing to uncover both known and novel mechanisms of drug resistance in MDR-MTB.

## Introduction

*Mycobacterium tuberculosis* (MTB), the causative agent of human tuberculosis (TB), was responsible for an estimated 10.7 million new cases and 1.2 million deaths worldwide in 2024, according to the World Health Organization (2025). While primarily a pulmonary infection, TB can also disseminate to extrapulmonary sites, including lymph nodes, pleura, and the brain (Pai et al., 2016). Based on single-nucleotide polymorphism (SNP) barcoding, the MTB complex is classified into seven major lineages (L1–L7) (Stucki et al., 2016). Among these, Lineage 2, particularly prevalent across Asia, has been frequently associated with heightened transmission, virulence, and drug resistance (Gagneux, 2018). The relentless emergence of drug-resistant MTB strains severely threatens global TB control efforts and jeopardizes the goal of ending the TB epidemic.

Globally, approximately 390,000 individuals were diagnosed with multidrug- or rifampicin-resistant TB (MDR/RR-TB) in 2024 (World Health Organization, 2025). MDR-TB is defined as resistance to the first-line drugs rifampicin (RIF) and isoniazid (INH). Pre-extensively drug-resistant TB (pre-XDR-TB) denotes MDR/RR-TB with additional resistance to any fluoroquinolone, while extensively drug-resistant TB (XDR-TB) meets the criteria for pre-XDR-TB plus resistance to at least one of bedaquiline (BDQ) or linezolid (LZD) (Dheda et al., 2024). The treatment success rate for MDR/RR-TB patients stands at only 68%, starkly lower than the 88% cure rate for drug-susceptible disease (World Health Organization, 2025; Dheda et al., 2024). This disparity is largely attributable to the frequent detection of resistance only after the failure of empirical therapy. Consequently, expeditious, mechanism-based precise diagnosis and the subsequent selection of appropriate therapeutic regimens are paramount to the success of the WHO’s End-TB strategy (World Health Organization, 2025).

Genome-wide association studies have successfully linked most resistance to first- and second-line anti-TB drugs to SNPs or short insertions and deletions (indels) within a finite set of chromosomal genes. However, short-read whole-genome sequencing (WGS) technologies systematically struggle to accurately resolve repetitive genomic loci, large indels, and mobile genetic element insertions, often leading to their misassembly or complete omission (Meehan et al., 2019). Long-read sequencing technologies have emerged as a powerful alternative to short-read next-generation sequencing (NGS), offering new insights into genetic architecture (Gómez-González et al., 2022; Zhao et al., 2023; Tang et al., 2024; Shi et al., 2025). Recent proof-of-concept studies showed their unique utility for the comprehensive analysis of structural variants (SVs), a class of genetic alterations (≥50 bp) that includes insertions, deletions, tandem duplications, inversions, and translocations, challenging for short-read platforms (Peker et al., 2021).

Very recently, a landmark study by Canalda-Baltrons et al. leveraged long-read assemblies from 859 diverse MTB isolates to construct a pangenome reference graph, revealing 3,077 SVs genome-wide and identifying multiple SV associations with drug resistance across 41,134 clinical isolates (Canalda-Baltrons et al., 2025). This work has substantially advanced our understanding of the landscape of SVs in MTB evolution and resistance. However, the functional validation of these newly identified, clinically relevant SVs, particularly unclassified variants within WHO-catalogued resistance genes, remains a critical gap. Large-scale association studies, while powerful for discovery, cannot by themselves establish causality or elucidate mechanistic pathways, which are essential for translating genomic findings into diagnostic and therapeutic applications.

In this study, we performed a comparative genomic analysis of clinical MDR-MTB isolates using both long-read (Oxford Nanopore Technologies, ONT) and short-read (Illumina) sequencing platforms. We employed two complementary computational frameworks, BCFtools and SVIM-asm, to generate a high-confidence map of MDR-associated SVs. Crucially, rather than performing another large-scale SV cataloging effort, we focused on two unclassified SVs that were uniquely identified in our MDR isolates and fell within WHO candidate resistance genes. Through a combination of Sanger sequencing, phenotypic susceptibility testing (pDST), heterologous expression in *Mycobacterium smegmatis* (*M. smegmatis*), minimum inhibitory concentration (MIC) determination, and AlphaFold 3-based structural modeling, we provide functional validation and mechanistic insight into these specific SVs.

## Materials and methods

### Bacterial culture

Eleven clinical MTB isolates (three susceptible and eight MDR) were collected from distinct patients diagnosed with TB at Wenzhou Central Hospital between July 2023 and June 2024. All patients were from the Wenzhou region of Zhejiang Province, China. The isolates were selected based on their phenotypic drug susceptibility profiles to include both drug-susceptible and MDR strains, with the MDR group further enriched for isolates showing resistance to second-line drugs. The MTB H37Rv reference strain was kindly provided by the National Tuberculosis Reference Laboratory at the Chinese Center for Disease Control and Prevention (Beijing, China). All bacterial strains were cultured in liquid medium using the BACTEC MGIT 960 system (Becton Dickinson, Franklin Lakes, NJ), and routine sub-culturing was performed to increase the bacterial load (>100 growth units, GU) and minimize contaminating human DNA and other flora.

### pDST assay

In accordance with the Clinical and Laboratory Standards Institute (CLSI) guidelines, pDST was performed for all antimicrobial agents using the BACTEC MGIT 960 system. Isolates were classified as resistant if growth in the drug-containing tube exceeded 1% of the growth in the drug-free control, as per CLSI criteria. No isolates were classified as borderline or intermediate resistance. The following 11 antibiotics were tested at the indicated critical concentrations: RIF (1 μg/mL), INH (0.1 μg/mL), ethambutol (EMB, 5 μg/mL), streptomycin (SM, 1 μg/mL), amikacin (AMK, 1 μg/mL), levofloxacin (LFX, 1 μg/mL), moxifloxacin (MFX, 0.25 μg/mL and 1 μg/mL), BDQ (1 μg/mL), LZD (1 μg/mL), pyrazinamide (PZA, 100 μg/mL) and ethionamide (ETO, 5 μg/mL). As PZA and ETO were not routinely tested in this laboratory, initial pDST was limited to the first nine antibiotics, and after genotypic predictions, supplementary pDST covering all 11 antibiotics was conducted.

### DNA extraction

Genomic DNA was extracted from MTB cultures using a protocol combining mechanical disruption via liquid nitrogen grinding with chemical lysis using sodium dodecyl sulfate (SDS), followed by purification with magnetic beads. Bacterial cells were firstly heat-inactivated by incubation at 80 °C for 10 min. Approximately 8 mL of culture was transferred to a 15 mL tube and centrifuged at 4,500 rpm for 10 min to pellet the cells. After washing with 1 × phosphate-buffered saline (PBS), the pellet was then homogenized in liquid nitrogen using a bead mill (50 Hz, 30 s; repeated twice). The resulting bacterial powder was lysed in 8 mL of SDS buffer at 50 °C for 1.5 h with intermittent mixing. Following centrifugation of the lysate (10,000 × g, 10 min), the supernatant was collected, and nucleic acids were extracted with an equal volume of phenol: chloroform: isoamyl alcohol (25: 24: 1, v/v), followed by extraction with chloroform: isoamyl alcohol (24: 1, v/v). DNA was precipitated from the aqueous phase by adding 0.8 volumes of ice-cold isopropanol, washed twice with 75% ethanol, and finally dissolved in Elution Buffer (EB). Contaminating RNA was removed by digestion with RNase A at 37 °C for 30 min. For further purification, AMPure XP magnetic beads (Beckman Coulter, Brea, CA) were used at a 0.5 × sample-to-bead ratio. The bead-bound DNA was washed twice with 75% ethanol and eluted in EB buffer. Final DNA quality assessment was performed using a NanoDrop spectrophotometer and agarose gel electrophoresis. DNA concentration was accurately determined using a Qubit dsDNA HS Assay Kit and measured on a Qubit 4.0 fluorometer (Thermo Fisher Scientific, Waltham, MA).

### WGS

For Nanopore long-read sequencing, libraries were prepared using the Ligation Sequencing Kit SQK-NBD114.96 (ONT, Oxford, UK). Briefly, DNA was end-repaired and dA-tailed using the NEBNext FFPE DNA Repair Mix (New England Biolabs, Ipswich, MA) and the NEBNext Ultra II End Repair/dA-Tailing Module (New England Biolabs). This was followed by ligation of native barcodes using the Native Barcoding Kit (ONT) and the Blunt/TA Ligation Master Mix (New England Biolabs). The barcoded libraries were pooled and adapter-ligated using the Native Barcoding Auxiliary Kit V14 (ONT). Prior to each enzymatic reaction, the DNA was purified using 1 × AMPure XP beads, washed twice with 80% ethanol, and eluted in EB. The final purified library was loaded onto a FLO-PRO114M (R10.4.1) flow cell (ONT). Sequencing was performed on a PromethION P48 instrument (ONT) controlled by MinKNOW software for approximately 48 h. The sequencing run was carried out by Benagen Technology (Hubei, China).

For Illumina short-read sequencing, libraries were prepared using the VAHTS Universal Plus DNA Library Prep Kit for Illumina V2 (Vazyme, Nanjing, China), following the manufacturer’s protocol. The final libraries were quantified and qualified before being sequenced on an Illumina NovaSeq X Plus platform (Illumina, San Diego, CA) to generate 151 bp paired-end reads.

### Pre-processing of the sequencing data

Raw Nanopore data, generated in real-time by MinKNOW and stored in FAST5 format, were base-called using Guppy v5.0.16 in super-accurate (SUP) mode and subsequently converted to FASTQ format (Wick et al., 2017a). To ensure high data quality, reads with a length shorter than 1,600 bp or a mean Phred quality score below 10 were filtered out using fastp v0.23.2 (Chen et al., 2018). Raw paired-end Illumina sequencing data were processed and quality-filtered using fastp v0.23.2. Read pairs were discarded if either read contained more than 10% undefined bases (‘N’) or if over 50% of the bases in either read had a Phred quality score (Q) ≤ 5.

Sequencing depth was calculated for both platforms by aligning the processed reads to the MTB H37Rv reference genome (GenBank accession no. NC_000962.3). Minimap2 v2.28 was employed for Nanopore long-read alignments (Li, 2018), while Bowtie 2 v2.5.4 was used for the Illumina short-read alignments (Langmead and Salzberg, 2012).

### *De novo* assembly of the MTB genomes

For long-read assembly, Nanopore long-read sequencing data were assembled *de novo* using Flye v2.9 (Lin et al., 2016). For short-read assembly, Illumina sequencing data were assembled *de novo* using Unicycler v0.5.0 (Wick et al., 2017b). For hybrid assembly, Flye v2.9 was first used to generate draft genomes from the Nanopore long reads, and these draft assemblies were then polished using the Illumina short-read data with Pilon v1.24 to correct small errors and improve base accuracy (Walker et al., 2014).

The quality of the three different assembly strategies (short-read-only, long-read-only, and hybrid) for each of the 12 samples was assessed using QUAST v5.3.0 (Mikheenko et al., 2023). The MTB H37Rv genome (NC_000962.3) was used as a reference, and key metrics evaluated included genome fraction (%), number of mismatches per 100 kbp, and number of insertions/deletions (indels) per 100 kbp. The circular genome map was generated using the R package circlize v0.4.18.

### Detection of anti-TB drug-resistance mutations

For the primary analysis, variants were identified by aligning the ONT-assembled genomes to the MTB H37Rv reference genome using Minimap2 v2.28 (Li, 2018). The resulting alignments were processed using SAMtools v1.21, and variant calling was performed with BCFtools v1.21 (Danecek et al., 2021), which is capable of detecting SVs larger than 50 bp. The called variants were annotated for their functional effects using snpEff v5.2 (Cingolani et al., 2012), which also generated annotations for assembled genomes.

To evaluate the impact of polishing, we also performed a parallel analysis using hybrid (ONT + Illumina) assemblies, following the same alignment and variant calling pipeline. The high concordance (>90%) between ONT-only and hybrid calls, particularly for the SVs of interest, confirmed the robustness of ONT-only SV detection.

Genotypic drug susceptibility testing (gDST) was performed by comparing the annotated variant results against the WHO’s “Catalogue of mutations in *Mycobacterium tuberculosis* complex and their association with drug resistance” (2nd edition) (World Health Organization, 2023). Variant positions and corresponding genes were matched against the catalogue to determine the associated drugs and the confidence grade of the association. The compiled gDST predictions were then used to classify isolates as MDR, pre-XDR, or XDR based on established definitions.

### Independent SV analysis with SVIM-asm

To cross-validate the SV calls from BCFtools, we performed an independent analysis using SVIM-asm v1.0.3 (Heller and Vingron, 2020), a SV caller specifically designed for haploid genome assemblies. The 12 *de novo* assembled genomes (both ONT-only and hybrid) were aligned pairwise to the H37Rv reference genome using Minimap2 v2.28 with the -x asm5 preset. The resulting alignment files (PAF format) were analyzed by SVIM-asm to detect SVs, including deletions, insertions, tandem duplications, and inversions. Only SVs ≥ 50 bp and supported by both BCFtools and SVIM-asm were retained for downstream analysis, ensuring a high-confidence SV set.

### Phylogenetic analysis

Lineage assignment was performed using the SNP-based classification scheme described by Stucki et al. (2016). Core SNPs were extracted from the ONT-assembled genomes using Snippy v4.6.0 (Seemann, 2015), retaining sites variable in at least one isolate. A maximum-likelihood phylogenetic tree was inferred from this alignment using IQ-TREE v2.3.6 with the following parameters: -m MFP to automatically determine the best-fit nucleotide substitution model via ModelFinder, and -bb 1,000 to calculate ultrafast bootstrap support values with 1,000 replicates (Minh et al., 2020). The tree was rooted on H37Rv.

### Verification of mutations by sanger sequencing

Specific primers were designed to flank the genomic regions containing the candidate mutations selected for validation. PCR amplification was performed using standard protocols. The resulting amplicons were purified and subjected to Sanger sequencing bidirectionally using the ABI PRISM 3730xl DNA Sequencer (Applied Biosystems, Foster City, CA) at Saiheng Biotechnology (Shanghai, China). The sequencing chromatograms were analyzed and compared to the MTB H37Rv reference genome to confirm the presence of the mutations.

### Construction of overexpressing strains

The MTB H37Rv genomic DNA was used as a template to amplify the wild-type *Rv0678* and *ethA* genes. The SV-type *Rv0678* gene (c.59_60ins1358 bp) was amplified from the genomic DNA of MDR clinical isolate R8, and the SV-type *ethA* gene (c.135_265del131 bp) from isolate R6. The His-tag was placed at the N-terminus of both the wild-type and SV-type constructs. The constructs were generated by ligating the PCR products into the pMV261 shuttle vector via BamHI and HindIII sites, followed by transformation into *E. coli* DH5α and verification by PCR and Sanger sequencing. The recombinant pMV261_Rv0678_WT, pMV261_Rv0678_SV, pMV261_ethA_WT and pMV261_ethA_SV plasmids were electroporated into *M. smegmatis mc^2^155* cells (abbreviated as Ms_Rv0678_WT, Ms_Rv0678_SV, Ms_ethA_WT, and Ms_ethA_SV), and the transformants were selected on Middlebrook 7H10 agar plates containing kanamycin (25 μg/mL), as suggested by Tang et al. (2025).

### Real-time quantitative PCR (RT-qPCR)

Total RNA was extracted from bacterial cells using the Trizol method, as described by Ma et al. (2024). The bacterial cells were cultured and harvested by centrifugation at 4 °C, 12,000 rpm for 5 min. The cell pellet was resuspended in 1 mL of Trizol reagent and mixed by vigorous vortexing for 15 s. After incubation at room temperature for 5 min, 200 μL of chloroform was added, and the mixture was shaken vigorously for 15 s, followed by incubation at room temperature for 10 min. The mixture was then centrifuged at 12,000 rpm for 15 min at 4 °C. The aqueous phase was transferred to a fresh RNase-free centrifuge tube, and an equal volume of isopropanol was added to precipitate the RNA. The sample was incubated at −20 °C for 2 h and then centrifuged at 12,000 rpm for 15 min at 4 °C. The RNA pellet was washed twice with 1 mL of 75% ethanol, air-dried, and dissolved in RNase-free water. Residual genomic DNA was removed, and cDNA was synthesized using the Evo M-MLV RT Premix for qPCR (Vazyme) according to the manufacturer’s instructions. Real-time quantitative PCR was performed using the SYBR Green Premix Pro Taq HS qPCR Kit (Vazyme). The reaction mixture (10 μL) consisted of 5 μL of 2 × SYBR Green Pro Taq HS Premix, 0.2 μL each of forward and reverse primers (10 μM), 2 μL of cDNA, and 2.6 μL of RNase-free ddH₂O. The qPCR was run on a real-time PCR system under the following conditions: 95 °C for 30 s, followed by 40 cycles of 95 °C for 5 s and 60 °C for 30 s. The 2^(−ΔΔCT) method was used to calculate the relative gene expression levels of *Rv0678* and *ethA*. The primer sequences used for RT-qPCR were as follows: *Rv0678* forward, 5′-ATGCGGGATCTGTTGGCATA-3′ and reverse, 5′-TCAGTCGTCCTCTCCGGTTC-3′; *ethA* forward, 5′-GCTGGAATCTCCGGTGTCAG-3′ and reverse, 5′-GCTTTGGATGTGAACGGTCC-3′.

### Western blotting

The heterologous expression strains (Ms_Rv0678_WT, Ms_Rv0678_SV, Ms_ethA_WT, Ms_ethA_SV), together with wild-type Ms (Ms_WT) and empty vector control (Ms_vec) strains, were cultured to mid-logarithmic phase (OD600 = 0.8–1.0). Bacterial cells were harvested by centrifugation at 8,000 rpm for 10 min at 4 °C and washed twice with PBS. The cell pellets were resuspended in PBS and lysed using a bacterial disruptor (15 cycles of 30 s each, with 30 s cooling on ice between cycles). The lysates were centrifuged at 12,000 rpm for 30 min at 4 °C, and the supernatants were collected. Loading buffer was added to the samples, followed by boiling at 100 °C for 10 min. Proteins were separated by 12% SDS-PAGE and transferred onto PVDF membranes. The membranes were blocked with 5% skim milk for 1 h at room temperature and then incubated with mouse anti-His-tag monoclonal antibody (1: 10,000 dilution) for 2 h at room temperature. After washing three times with TBST, the membranes were incubated with HRP-conjugated goat anti-mouse IgG secondary antibody (1: 2,000 dilution) for 1 h at room temperature. Protein bands were visualized using an ECL chemiluminescence kit (Thermo Fisher Scientific) and imaged with a chemiluminescence imaging system (Tanon Science & Technology, Shanghai, China).

### Growth curve

The *M. smegmatis* (Ms), *M. smegmatis* with pMV261 (Ms_vec), Ms_Rv0678_WT, Ms_Rv0678_SV, Ms_ethA_WT, and Ms_ethA_SV strains at the logarithmic phase were inoculated into Middlebrook 7H9 medium (Becton Dickinson, Franklin Lakes, NJ) at an initial OD600 of 0.01 and cultured in a shaker at 37 °C, 250 rpm. The growth of the strains was monitored by measuring OD600 every 4 h for up to 78 h using a spectrophotometer.

### MIC determination

The minimum inhibitory concentrations (MICs) of BDQ and ETO against the heterologous expression strains were determined by the microplate-based method, with 100 μL of the bacterial suspension added to each well. The strains were cultured in Middlebrook 7H9 medium to logarithmic growth phase, and the bacterial suspension was adjusted to approximately 2 × 10^5^ CFU/mL. Two-fold serial dilutions of antibiotics were prepared in 96-well plates. For BDQ, the final concentrations ranged from 0.016 to 8 μg/mL. For ETO, the final concentrations ranged from 1 to 512 μg/mL. Bacterial growth was assessed by measuring OD600 after 4 days of incubation at 37 °C. All experiments were performed in triplicate.

### Prediction of protein tertiary structure by AlphaFold 3

The wild-type and mutant amino acid sequences of Rv0678 and EthA proteins were retrieved from the annotated genomic data. These sequences were submitted to the AlphaFold Server^1^ for protein tertiary structure prediction using AlphaFold 3 (Abramson et al., 2024). The resulting predicted structures were visualized and analyzed to assess the structural consequences of the frameshift mutations.

### Statistical analysis

Continuous variables with skewed distributions are presented as medians with interquartile ranges and compared using the Mann–Whitney U test (GraphPad Prism version 8.0.2), with multiple comparisons corrected via the Benjamini method. Statistical significance was also assessed for MIC data using two-way repeated-measures ANOVA with Sidak’s multiple comparisons test (GraphPad).

## Results

### Resistance profiles of clinical MTB isolates and the H37Rv strain

The MTB H37Rv reference strain, three susceptible clinical isolates (S2-S4), and 8 MDR clinical isolates (R1-R8) were included in this study (Figure 1a; Supplementary Table S1). The H37Rv strain, which is fully susceptible, served as an internal control throughout the experimental workflow. pDST against a panel of nine anti-TB drugs confirmed that all 8 MDR isolates (R1–R8) were resistant to at least RIF and INH (Figure 1b). Notably, isolates R2–R6 and R8 exhibited additional resistance to LFX and MFX, meeting the WHO definition for pre-XDR-TB. After genotypic predictions, supplementary pDST was performed for PZA and ETO, which confirmed resistance profiles fully consistent with the mutations identified in the corresponding resistance genes (Figure 1c).

**Figure 1:**
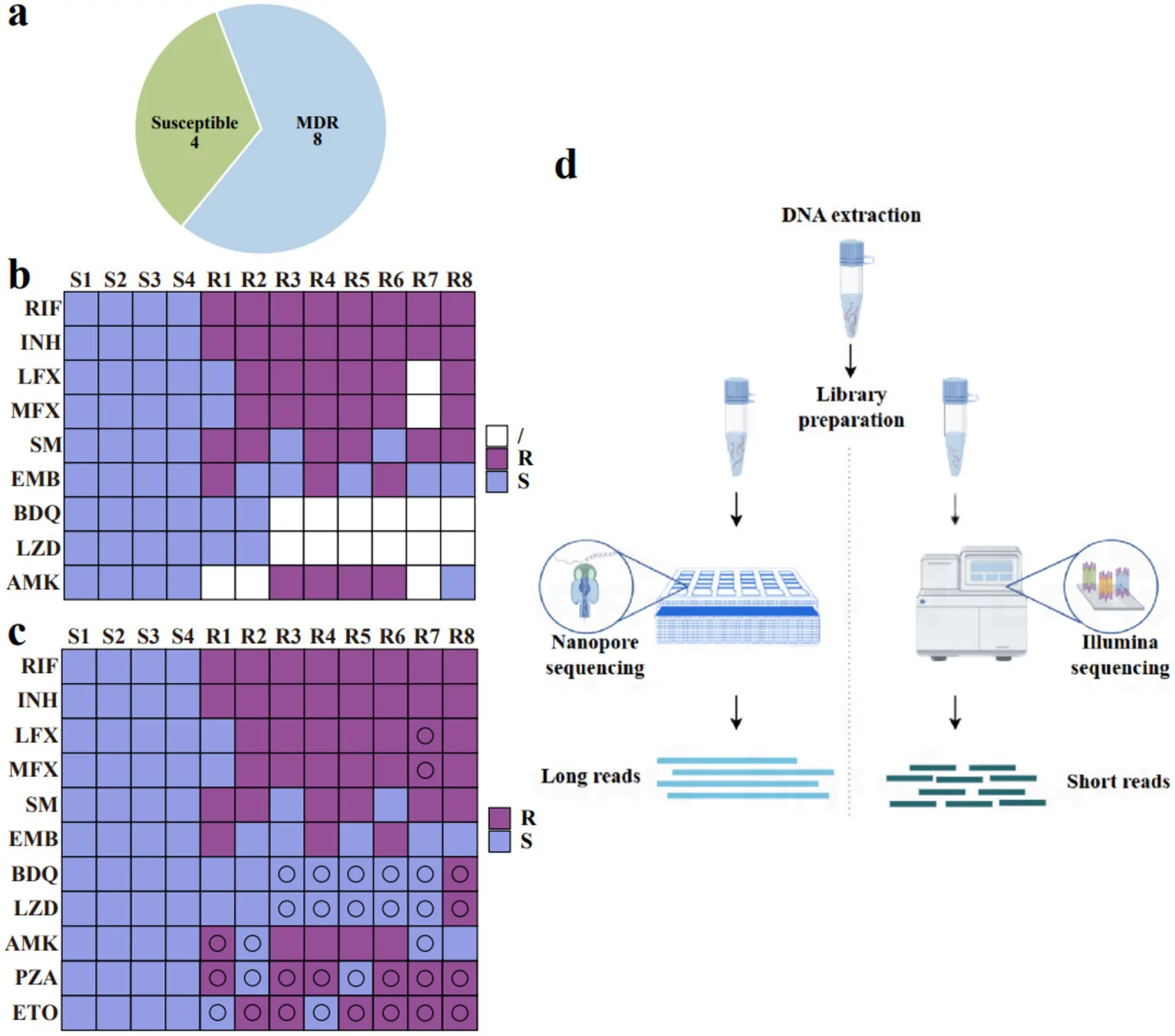
Drug resistance profiles and genomic sequencing workflow of clinical *Mycobacterium tuberculosis* (MTB) isolates plus the MTB H37Rv reference strain **(a)** The H37Rv strain (S1), three susceptible MTB isolates (S2-S4), and eight multidrug-resistant MTB isolates (R1-R8), were included in this study. **(b)** Initial phenotypic drug susceptibility profiles against the nine routinely tested anti-tuberculosis drugs. **(c)** Supplementary phenotypic drug susceptibility profiles, obtained after genotypic prediction. Circles (Ο) indicate additional phenotypic resistance data. **(d)** Schematic workflow of the whole-genome sequencing (WGS). Genomic DNA was extracted and subjected to library preparation and WGS on both Oxford Nanopore Technologies (ONT) and Illumina platforms. S, susceptible; R, multidrug-resistant; /, not tested. RIF, rifampicin; INH, isoniazid; LFX, levofloxacin; MFX, moxifloxacin; SM, streptomycin; EMB, ethambutol; BDQ, bedaquiline; LZD, linezolid; AMK, amikacin; PZA, pyrazinamide; ETO, ethionamide.

### Long-read sequencing generated near-complete and highly contiguous MTB genomes

As illustrated in Figure 1d, genomic DNA from all cultures was subjected to WGS on both ONT (R10.4.1 flow cells) and Illumina platforms. A bioinformatics workflow was established for comparative analysis (Figure 2a). Both platforms yielded high-quality data with substantial depth (>100×; Figure 2b, Supplementary Table S2, S3). ONT-only assemblies covered 99.14% of the H37Rv reference genome on average, with a mean of only 2.17 contigs, demonstrating superior contiguity compared to Illumina-only assemblies (mean 132.5 contigs; Figure 2c–e; Supplementary Tables S4–S7). Polishing the ONT assemblies with Illumina short reads did not significantly improve genome fraction coverage (Figure 2c), and the numbers of mismatches and indels per 100 kbp were comparable between ONT-only and hybrid approaches (Figure 2d). Consistent with recent benchmarks and our previous studies (Tang et al., 2024; Shi et al., 2025), the R10.4.1 flow cell chemistry provided sufficient base-level accuracy for reliable variant calling, including SVs. A representative near-complete circular genome map is shown in Figure 2f.

**Figure 2:**
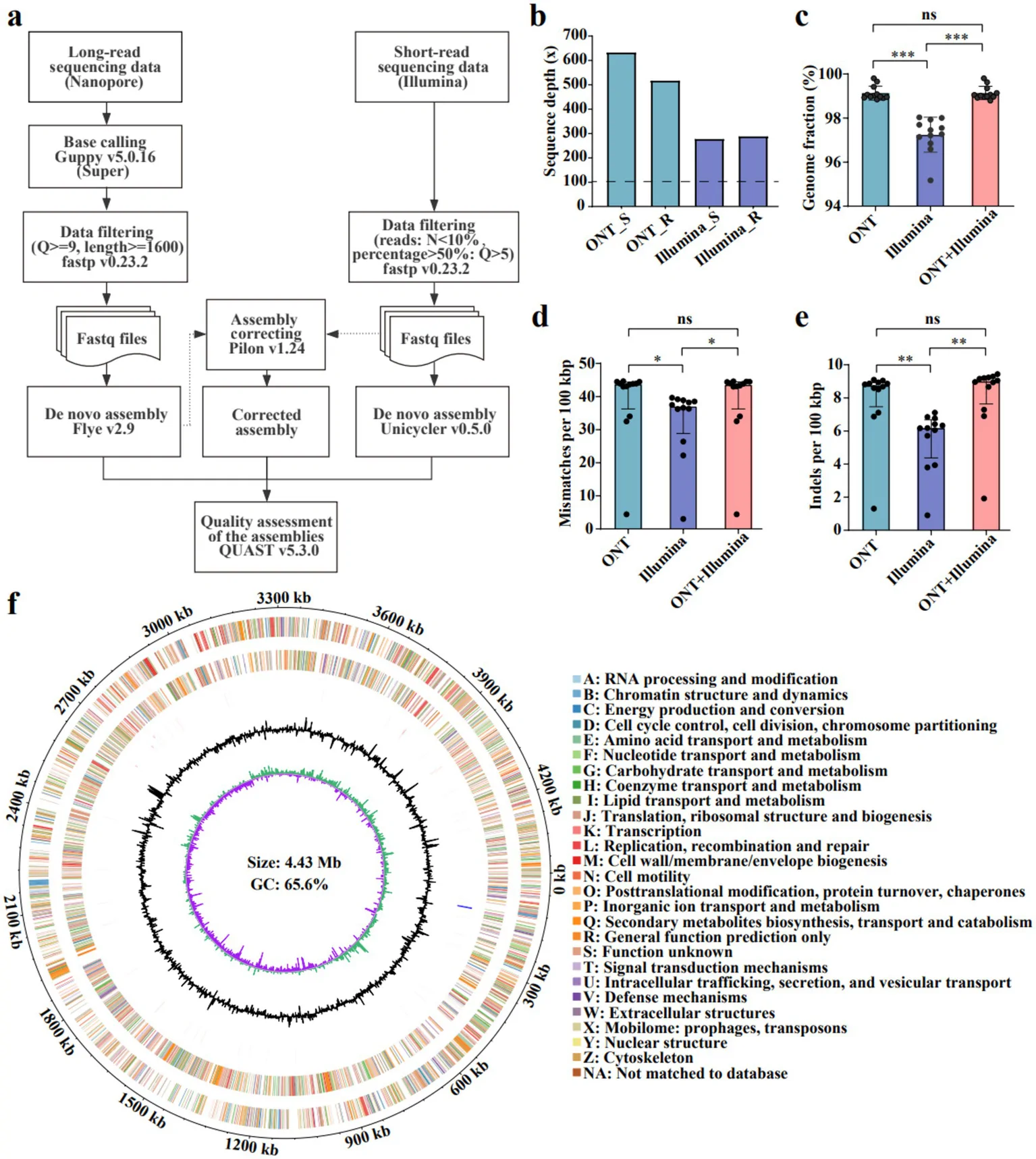
Quality assessment of sequencing data and *de novo* genome assembly of *Mycobacterium tuberculosis* (MTB). **(a)** Bioinformatics pipeline for processing ONT long-read and Illumina short-read sequencing data. **(b)** Average sequencing depth (>100×) achieved for each group (S, Susceptible; R, Resistant) on the ONT and Illumina platforms. **(c–e)** Genome fraction (%) and average number of mismatches and insertions/deletions (indels) per 100 kbp of the MTB H37Rv reference genome covered by alignments of 12 assemblies, generated using ONT long-read, Illumina short-read and hybrid (ONT + Illumina) approaches. Mann–Whitney U test; ns, not significant; *, *p* < 0.05; **, *p* < 0.01; ***, *p* < 0.001. **(f)** Circular genome map of a representative near-complete MTB isolate assembled. The map displays the following tracks from outer to inner ring: (1) Genomic coordinates (Mb); (2) Protein-coding genes on the forward strand, colored by Clusters of Orthologous Groups (COG) functional categories; (3) Protein-coding genes on the reverse strand, similarly colored by COG categories; (4) Locations of ribosomal RNA (rRNA, blue) and transfer RNA (tRNA, red) genes; (5) GC content plot calculated in 2,000-bp sliding windows; (6) GC skew (G-C)/(G + C) plot (2,000-bp windows), with green and purple indicating regions where G > C and G < C, respectively.

### Identification of MDR-associated mutations and exclusive focus on unclassified SVs within WHO-catalogued resistance genes

To delineate genetic variants associated with MDR, we aligned ONT-assembled genomes to the H37Rv reference and performed variant calling using BCFtools. This analysis identified 3,233 mutations (including 132 SVs, comprising 83 insertions and 49 deletions) across 588 genes that were unique to the MDR isolates (Figure 3a). The overlapping regions in Figure 3a represent variant-containing genes shared between the MDR and susceptible groups, while the non-overlapping regions represent genes with variants exclusive to each group. Given the recent comprehensive SV catalog by Canalda-Baltrons et al. (2025), we did not pursue a genome-wide SV enumeration but instead focused on clinically actionable variants within the 65 WHO-catalogued resistance genes (Supplementary Table S8). Within this restricted gene set, we detected 88 mutations across 14 genes exclusively in MDR isolates, of which 65 were previously classified by the WHO catalogue and 23 were unclassified (Supplementary Table S9). Crucially, only two SVs fell within these WHO-catalogued genes: *Rv0678* c.59_60ins1358bp (p.Gln22fs) and *ethA* c.135_265del (p.Trp45fs) (Figure 3a, dotted circles). Hybrid (ONT + Illumina) assemblies yielded nearly identical results (89 mutations, including the same two SVs), with >90% concordance (Supplementary Figure S1; Supplementary Table S10), confirming the robustness of ONT-only SV detection.

**Figure 3:**
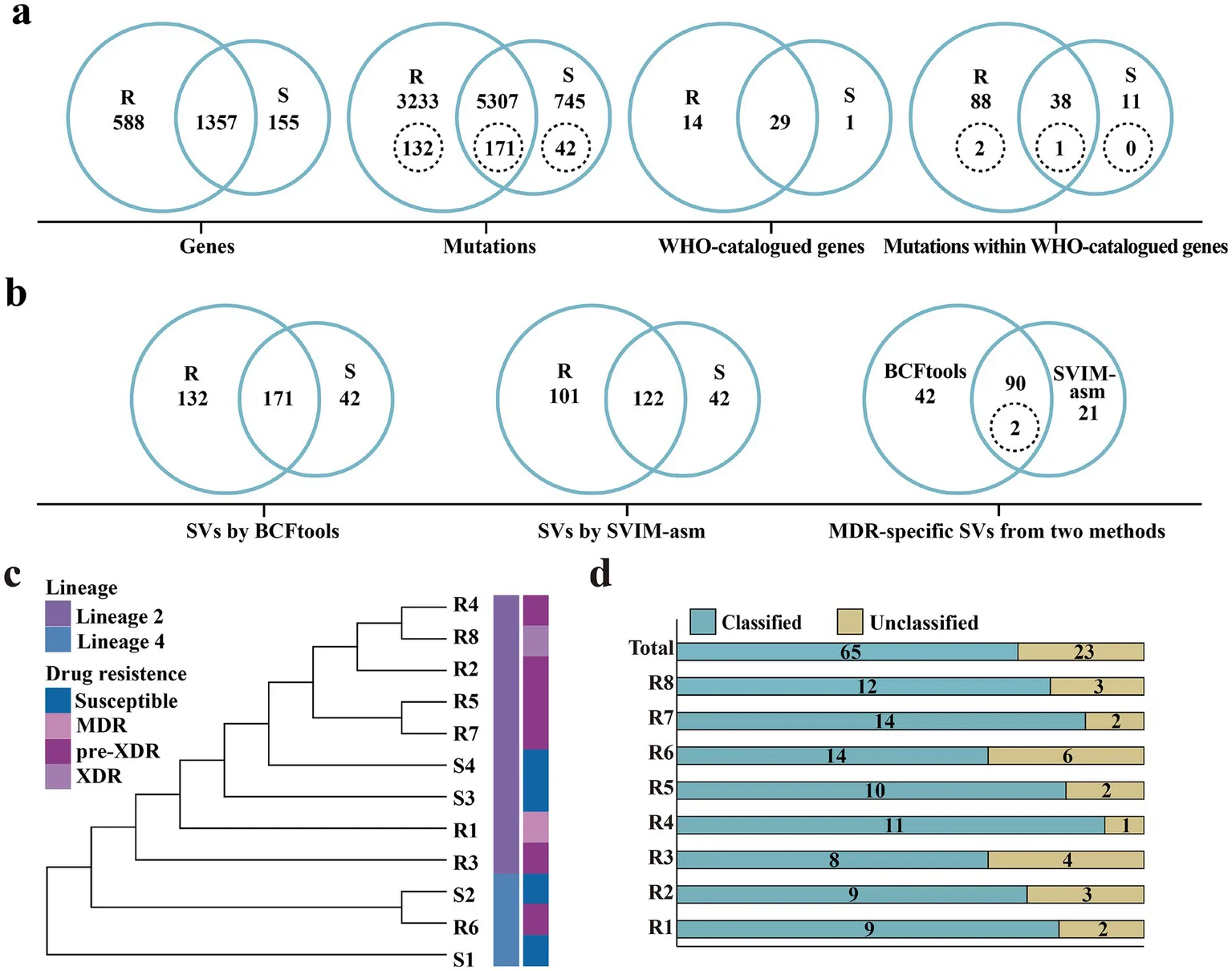
Distribution of genetic variants between multidrug-resistant (MDR) and susceptible *Mycobacterium tuberculosis* (MTB) isolates, with the H37Rv reference strain included in the susceptible group. **(a)** Overlap of variant-containing genes and individual variants identified at the whole-genome scale between the eight MDR and the three susceptible isolates plus H37Rv, using the ONT-only assembly approach. Genes harboring variants that also belong to the candidate drug resistance genes in the WHO catalogue are shown, together with the individual variants located within these catalogue genes. Dotted circles denote structural variants (SVs). **(b)** Overlap of SVs identified at the whole-genome scale by BCFtools and SVIM-asm between the MDR and susceptible groups, derived from ONT-only assemblies. Dotted circles highlight MDR-specific SVs that were detected by both callers and reside within candidate drug resistance genes of the WHO catalogue. **(c)** Maximum-likelihood phylogenetic tree of the 11 clinical isolates plus H37Rv, inferred from core single-nucleotide polymorphisms (SNPs) extracted from ONT-only assemblies. **(d)** Stacked bar chart showing the numbers of classified and unclassified mutations found exclusively in the MDR isolates within candidate drug resistance genes of the WHO catalogue, based on ONT-only assemblies.

To further validate SV specificity, we performed an independent SV call using SVIM-asm on the same assemblies. This method identified 101 MDR-specific SVs, with 90 overlapping those from BCFtools (Figure 3b). Notably, the only SVs consistently detected by both callers and located within WHO-catalogued resistance genes were precisely the *Rv0678* and *ethA* variants (Figure 3b, dotted circles). Phylogenetic analysis based on core SNPs confirmed that most clinical isolates belonged to Lineage 2 (Figure 3c; Supplementary Figure S1c), the dominant MTB clade circulating in China, which is consistent with the high prevalence of drug resistance in this lineage, and their mutations classified by the WHO catalogue were illustrated in Figure 3d and Supplementary Figure S1d.

### Sanger sequencing and pDST validated the two unclassified SVs

We selected six representative mutations for experimental validation by Sanger sequencing, including three classified variants (*pncA* c.528delC, *Rv0678* c.139dupG, *ethA* c.1114delA) and the three unclassified variants (*pncA* c.417_424dupGCGCCAGA, *Rv0678* c.59_60ins1358bp, *ethA* c.135_265del). The distribution of the six selected mutations across the studied strains and their lineage assignments was summarized in Supplementary Table S11. With *Rv0678* c.59_60ins1358bp and *ethA* c.135_265del SVs identified in a single MDR isolate (R8 and R6, respectively), the variants were distributed across multiple isolates, consistent with independent acquisition rather than a single clonal expansion. As reported, these variants were associated with resistance to PZA, BDQ/clofazimine and ETO, respectively (Zhang et al., 2014; Omar et al., 2022; Snobre et al., 2023; Vilchèze and Jacobs, 2014).

Sanger sequencing confirmed all six mutations (Figures 4a–f). Importantly, pDST results for the three classified variants matched their genotypic predictions (Figure 1b). For the three unclassified variants, supplementary pDST demonstrated that *pncA* c.417_424dupGCGCCAGA conferred PZA resistance, *Rv0678* c.59_60ins1358bp conferred BDQ resistance, and *ethA* c.135_265del conferred ETO resistance (Figure 1c). As suggested by the literature (Miotto et al., 2017), these data established a clear genotype–phenotype link for the two SVs, warranting further functional investigation.

**Figure 4:**
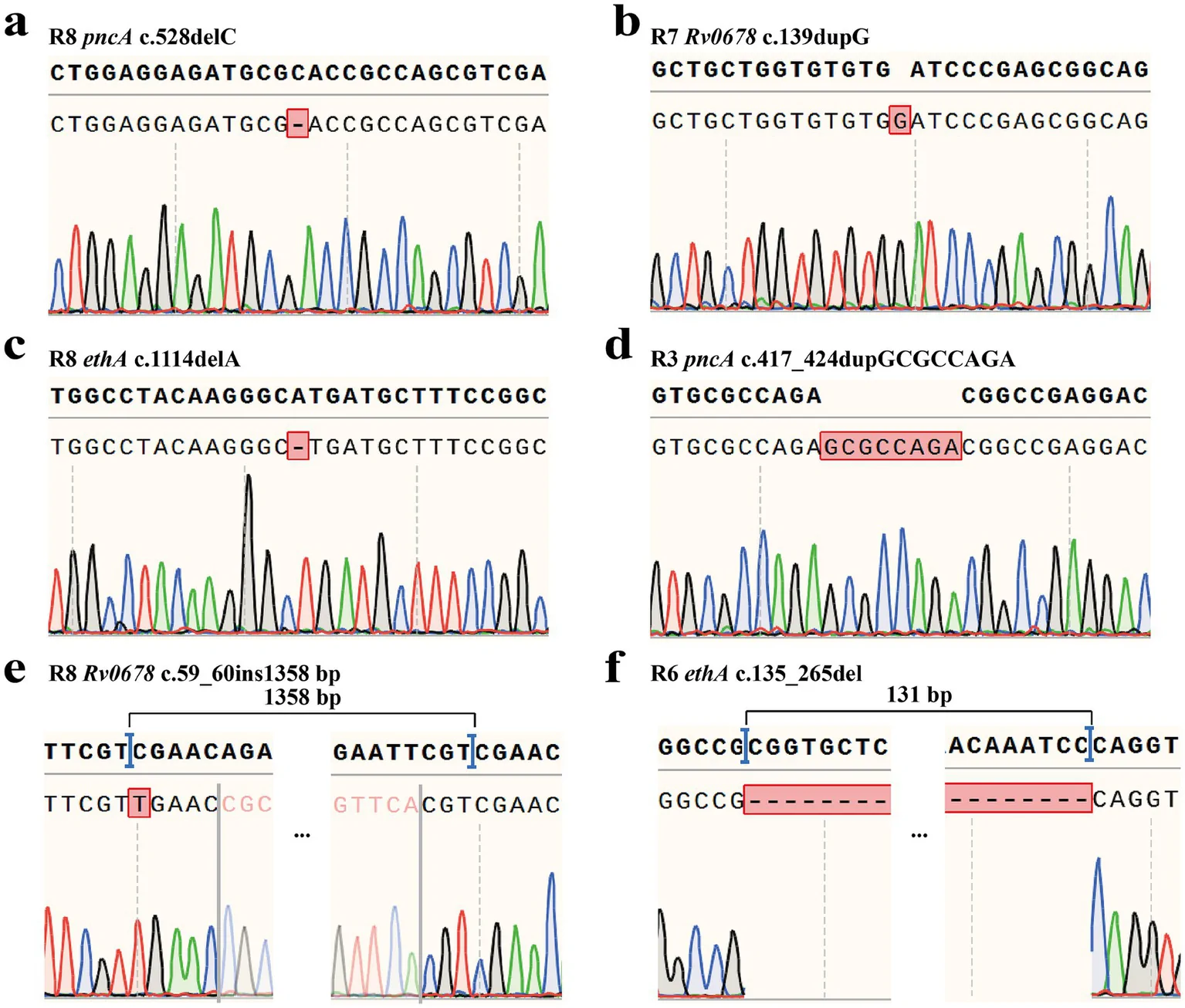
Sanger sequencing validation of mutations in *Mycobacterium tuberculosis* (MTB) isolates. **(a–f)** Sanger sequencing chromatograms of six selected mutation sites: **(a)** *pncA* c.528delC (p.Thr177fs); **(b)** *Rv0678* c.139dupG (p.Asp47fs); **(c)** *ethA* c.1114delA (p.Met372fs); **(d)** *pncA* c.417_424dupGCGCCAGA (p.Thr142fs); **(e)** *Rv0678* c.59_60ins1358bp (p.Gln22fs); and **(f)** *ethA* c.135_265del (p.Trp45fs). The *MTB* H37Rv reference sequence is shown above each chromatogram for mutant comparison, with the insertion/deletion site marked.

### Heterologous expression in *Mycobacterium smegmatis* confirmed that the two SVs confer drug resistance

*M. smegmatis* was selected as the heterologous host because it is a well-established surrogate for functional studies of mycobacterial genes, with a rapid growth rate and lower biosafety requirements compared with MTB (Sparks et al., 2023; Lelovic et al., 2020). Its EthA and Rv0678 homologues are sufficiently divergent from their MTB counterparts to permit functional discrimination of the introduced alleles.

We constructed recombinant *M. smegmatis* strains overexpressing wild-type or SV-type *Rv0678* and *ethA*. RT-qPCR confirmed robust overexpression of both genes in the respective heterologous expression strains (Figures 5a,b). To directly assess whether the frameshift mutations lead to truncated proteins, we performed Western blotting using anti-His-tag antibodies (Figures 5c, d). The wild-type strains produced full-length proteins of the expected sizes. However, in both Ms_Rv0678_SV and Ms_ethA_SV, the predicted truncated peptides were undetectable, which might be related to rapid degradation, lack of translation, or failure of the gel system to retain the peptide.

**Figure 5:**
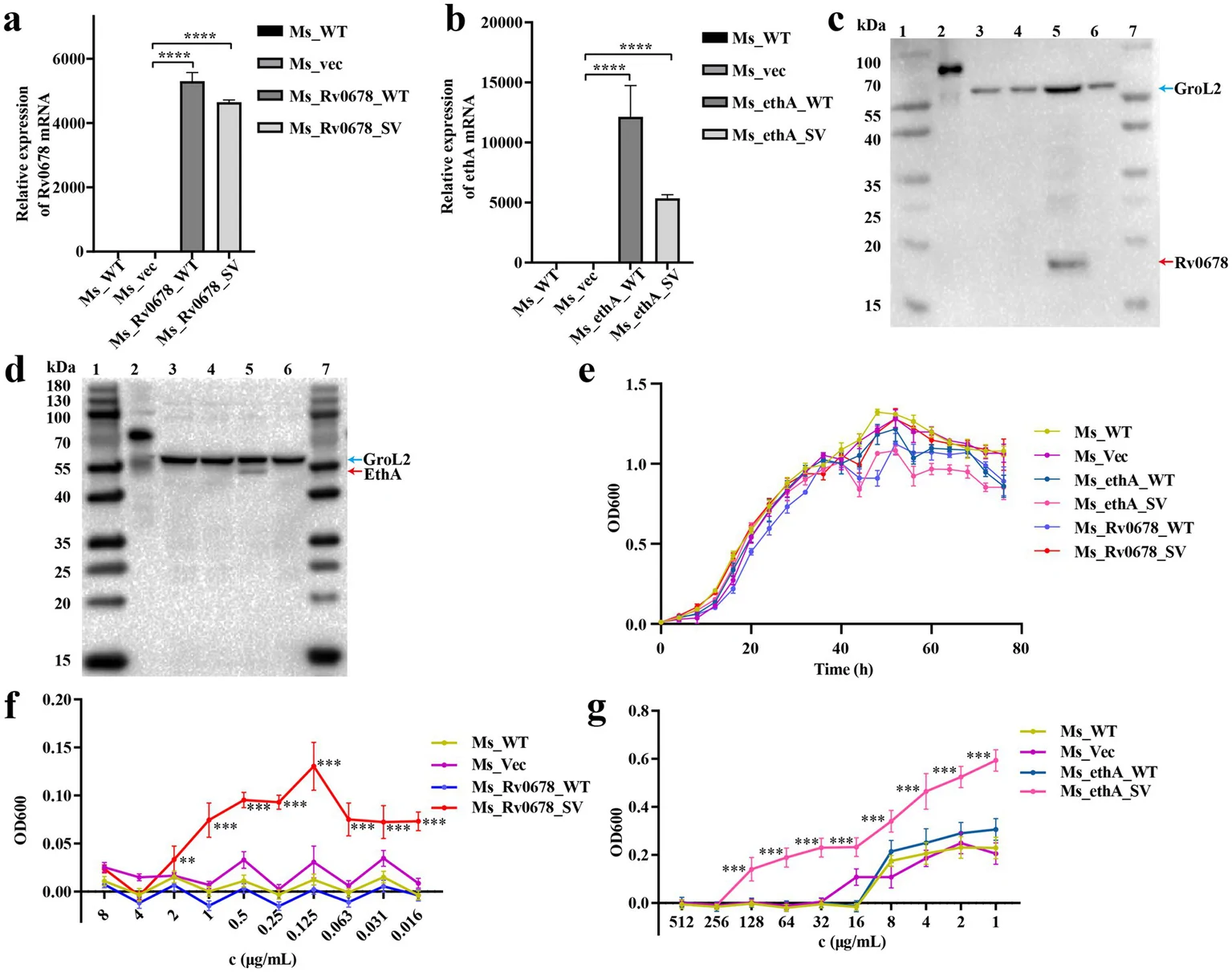
Construction and characterization of recombinant *Mycobacterium smegmatis* strains overexpressing wild-type and structural variant forms of *Rv0678* and *ethA* **(a,b)** RT-qPCR validation of *Rv0678* **(a)** and *ethA* **(b)** overexpression in heterologous expression strains **(c)** Western blot analysis of Rv0678 expression in heterologous expression strains. A band corresponding to wild-type Rv0678 (~18 kDa) was detected in Ms_Rv0678_WT (lane 5). In Ms_Rv0678_SV, the predicted truncated protein was not detectable. The GroL2 protein (56 kDa) with His-tag was endogenously expressed. Lane 1 and 7: Protein marker; lane 2: His-tagged positive control; lane 3: Ms_WT; lane 4: Ms_vec; lane 5: Ms_Rv0678_WT; lane 6: Ms_Rv0678_SV. Primary antibody: Anti-His-tag monoclonal antibody **(d)** Western blot analysis of EthA expression in heterologous expression strains. A band corresponding to wild-type EthA (~55 kDa) was detected in Ms_ethA_WT (lane 5). In Ms_ethA_SV, the predicted truncated protein was not detectable. Lane 1 and 7: Protein marker; lane 2: His-tagged positive control; lane 3: Ms_WT; lane 4: Ms_vec; lane 5: Ms_ethA_WT; lane 6: Ms_ethA_SV **(e)** Growth curves of wild-type and heterologous expression strains **(f,g)** Minimum inhibitory concentration (MIC) determination of bedaquiline (BDQ, f) and ethionamide (ETO, **g**) against heterologous expression strains. **, *p* < 0.01; ***, *p* < 0.001. WT, wild-type; vec, empty vector control.

All strains reached the plateau phase at 36 h with comparable cell densities, and no significant differences were observed among strains at this time point (Figure 5e), from which cells were harvested for subsequent analyses. Critically, MIC determination revealed that the BDQ MIC of Ms_Rv0678_SV was 4 μg/mL versus <0.016 μg/mL for controls (Figure 5f). Because <0.016 μg/mL is the lowest concentration tested, the fold-change is censored. Similarly, Ms_ethA_SV exhibited an ETO MIC of 256 μg/mL, which was 16-fold higher than that of Ms_ethA_WT (16 μg/mL) and 8-fold higher than that of Ms_vec (32 μg/mL) (Figure 5g). These results provided experimental evidence that the *Rv0678* c.59_60ins1358bp insertion and the *ethA* c.135_265del deletion conferred resistance to BDQ and ETO, respectively.

### AlphaFold 3-based structural predictions provided structural context for loss-of-function mutations

Using AlphaFold 3, a tool renowned for its high-accuracy predictions of protein structures and complexes (Abramson et al., 2024), we predicted the tertiary structures of wild-type and mutant Rv0678 and EthA proteins. For *Rv0678* c.59_60ins1358bp, the frameshift introduced a premature termination codon at position 22, resulting in a severely truncated protein lacking the C-terminal 44 amino acids, which constituted the putative DNA-binding repressor domain (Figures 6a–c), as suggested by the literature (Radhakrishnan et al., 2014). The most plausible mechanism is that the *IS6110* insertion abrogates production of a functional Rv0678 repressor, leading to constitutive upregulation of the MmpS5-MmpL5 efflux pump and reduced intracellular BDQ accumulation, ultimately resulting in the observed phenotypic resistance to BDQ (Figure 6d). For *ethA* c.135_265del, the frameshift deleted 131 bp and introduced a premature stop codon, producing a truncated EthA protein missing a substantial portion of the C-terminal domain (Figures 6e–g). As EthA is a monooxygenase required for activation of the pro-drug ETO, loss of its function prevents formation of the active ETO-NAD adduct that normally inhibits InhA (Vilchèze and Jacobs, 2014), thereby blocking mycolic acid biosynthesis and conferring resistance (Figure 6h).

**Figure 6:**
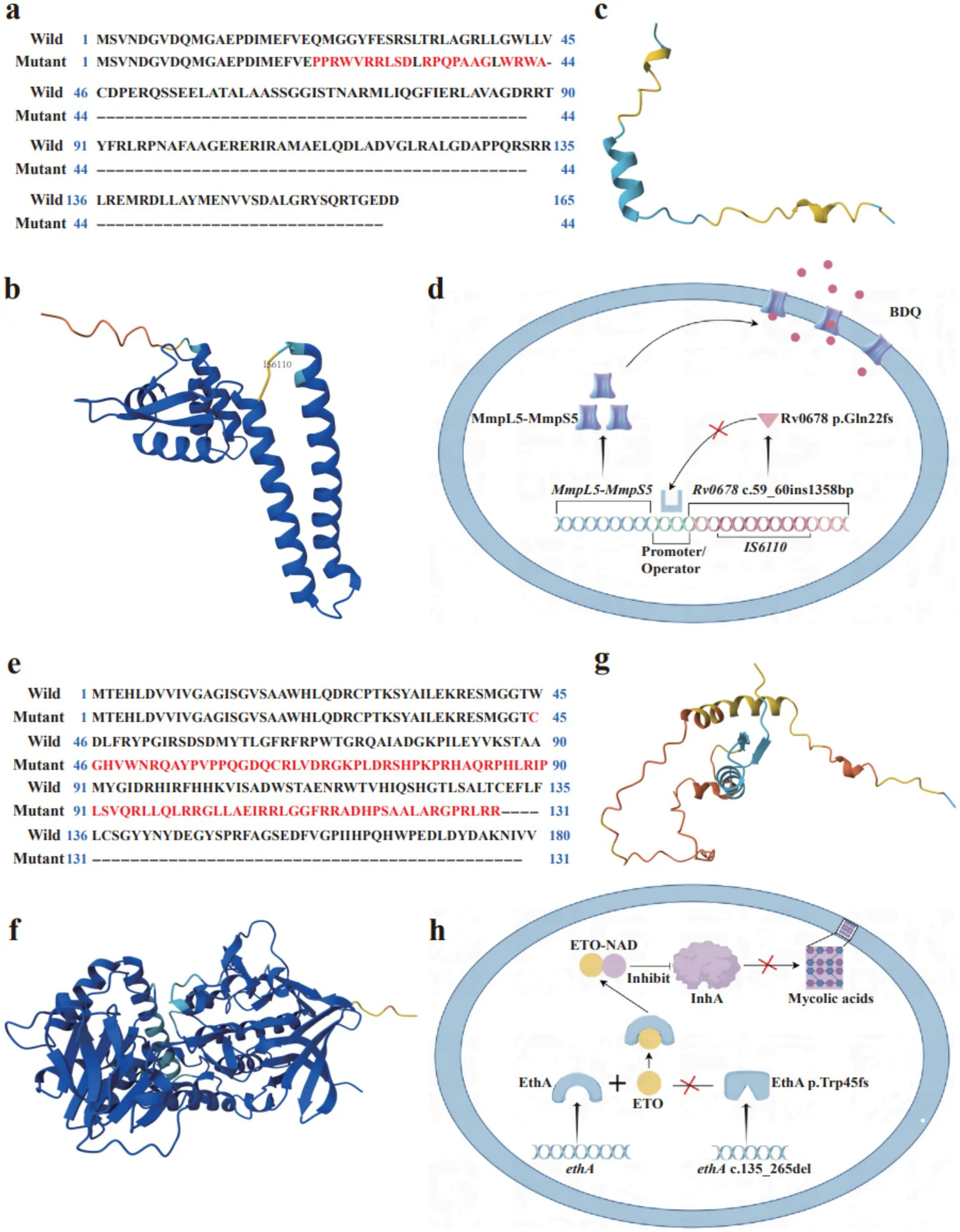
Molecular consequences of structural variants from *Rv0678* and *ethA* with proposed mechanisms of drug-resistance. **(a)** Schematic representation of wild-type and mutant Rv0678 protein sequences. The *IS6110* insertion site and the premature termination codon (PTC) introduced by the frameshift are indicated. **(b,c)** Predicted tertiary structures of the wild-type **(b)** and truncated mutant **(c)** Rv0678 proteins, generated using AlphaFold 3. The missing C-terminal domain in the mutant protein is highlighted. **(d)** Literature-based reference model of *Rv0678* c.59_60ins1358bp (p.Gln22fs) for bedaquiline (BDQ) resistance. Insertion of the *IS6110* mobile element into the *Rv0678* gene leads to a loss-of-function mutation. The resulting absence of the functional repressor protein causes dysregulation and constitutive upregulation of the MmpS5-MmpL5 efflux pump, thereby reducing intracellular BDQ concentration and conferring resistance. **(e)** Schematic representation of wild-type and mutant EthA protein sequences. The deletion site and the PTC introduced by the frameshift are indicated. **(f,g)** Predicted tertiary structures of wild-type **(f)** and truncated mutant **(g)** EthA proteins, generated using AlphaFold 3. The missing C-terminal domain in the mutant protein is highlighted. **(h)** Literature-based reference model of *ethA* c.135_265del (p.Trp45fs)for ethionamide (ETO) resistance. ETO is a pro-drug activated by EthA. The active form of ETO reacts with NAD^+^ to yield an ETO-NAD adduct, which inhibits the enoyl ACP reductase InhA, leading to inhibition of mycolic acid biosynthesis. The truncated EthA protein lacks the C-terminal monooxygenase domain essential for ETO activation, resulting in no inhibition of InhA and continued mycolic acid biosynthesis, thereby conferring ETO resistance.

## Discussion

The escalating threat of drug-resistant tuberculosis necessitates a deep understanding of its underlying genetic mechanisms to guide effective countermeasures (World Health Organization, 2025; Pai et al., 2016; Gagneux, 2018; Dheda et al., 2024). While short-read WGS has revolutionized the detection of resistance-conferring SNPs and short indels, its limitations in resolving repetitive regions and complex genomic alterations are well-documented (Meehan et al., 2019). In this study, by applying ONT long-read sequencing to a collection of clinical MDR-MTB isolates, we reliably called SNPs and, more importantly, accurately detected SVs that would have been systematically missed by short-read technologies (Gómez-González et al., 2022; Zhao et al., 2023; Tang et al., 2024; Shi et al., 2025; Peker et al., 2021; Canalda-Baltrons et al., 2025).

The two SVs we identified, *Rv0678* c.59_60ins1358bp (p.Gln22fs) and *ethA* c.135_265del (p.Trp45fs), were exclusively detectable by long-read sequencing and functionally validated through Sanger sequencing, pDST, heterologous expression in *M. smegmatis*, MIC determination, Western blotting, and AlphaFold 3-based structural modeling. These findings directly address a critical gap highlighted by the landmark study of Canalda-Baltrons et al. (2025), who constructed a pangenome reference graph from 859 long-read assemblies, identified 3,077 SVs genome-wide, and associated multiple SVs with drug resistance across 41,134 clinical isolates. However, as the authors themselves acknowledged, association studies alone cannot establish causality. Our study provides the functional validation that their large-scale discovery necessarily lacked, bridging the gap between population-level variant identification and individual-level mechanistic understanding.

The two SVs illustrate both the recurrent nature of certain resistance mechanisms and the allelic diversity that long-read sequencing can uncover. For *Rv0678*, *IS6110*-mediated disruption is an established mechanism of BDQ resistance, and there were five clinical isolates across 6,426 genomes carrying *IS6110* insertions in *Rv0678*, at positions 71 bp (in four isolates: TB_RSA126, TB_RSA64, TB_RSA63, KT-0084) and 76 bp (in TKK-01-0074), with three sharing an identical *IS6110* barcode suggestive of transmission (Antoine et al., 2021). The insertion we report, c.59_60ins1358bp, occurs at a different position (between codons 59 and 60), representing a new allelic variant of this established resistance mechanism (Antoine et al., 2021). Our functional validation, heterologous expression, MIC determination, and Western blotting, confirms that this specific allele, like its previously reported counterparts, leads to loss of the C-terminal repressor domain and consequent MmpS5-MmpL5 upregulation (Omar et al., 2022; Snobre et al., 2023). The AlphaFold 3-predicted structural model supports this loss-of-function mechanism by showing elimination of the C-terminal DNA-binding domain (Radhakrishnan et al., 2014).

For *ethA*, the c.135_265del deletion we identified is, to our knowledge, a novel SV. While ethA loss-of-function is a well-recognized mechanism of ETO resistance (Vilchèze and Jacobs, 2014), and large-scale surveys have documented *ethA* SVs clustering in the Beijing lineage (Canalda-Baltrons et al., 2025), the precise 131-bp deletion we report has not been previously described. Notably, this deletion lies entirely within the larger c.105_1760del region reported by Canalda-Baltrons et al. (2025) in 131 resistant isolates, suggesting that our finding represents a specific allelic form of a broader resistance-associated deletion class. The 16-fold increase in ETO MIC conferred by this deletion in *M. smegmatis*, together with the RT-qPCR and Western blotting results, provides functional evidence for its role in resistance. AlphaFold 3 predictions further provide a structural rationale for the loss of monooxygenase activity.

The integration of genotypic prediction with multi-layered phenotypic validation is the cornerstone of resistance mechanism discovery (Dheda et al., 2024; Miotto et al., 2017; Andries et al., 2005). In this study, recombinant *M. smegmatis* strains overexpressing the SV-type *Rv0678* or *ethA* exhibited significantly elevated MICs for BDQ (4 μg/mL vs. <0.016 μg/mL) and ETO (256 μg/mL vs. 16 μg/mL for wild-type), respectively. These results provide experimental evidence that these SVs confer resistance, and highlight the value of *M. smegmatis* as a rapid and safe surrogate for functional studies of MTB resistance alleles (Sparks et al., 2023; Lelovic et al., 2020).

Lineage 2 strains predominated in our collection, consistent with their epidemiological significance in China and their well-established association with drug resistance (Gagneux, 2018). Among the 88 mutations we identified within WHO-catalogued resistance genes that were unique to MDR isolates, 65 were previously classified and 23 were unclassified. Notably, most of the 90 MDR-specific SVs detected by both BCFtools and SVIM-asm fell outside the WHO candidate gene set. These findings underscore the complexity of the MDR-MTB resistome and highlight the need for continuous curation of resistance databases as the genetic determinants of resistance continue to evolve (Meehan et al., 2019; Tang et al., 2024).

Several limitations should be acknowledged. First, while our functional validation in *M. smegmatis* demonstrated that the *ethA* c.135_265del deletion conferred ETO resistance, this heterologous system does not fully recapitulate the native regulatory and metabolic context of MTB, and the endogenous orthologues MSMEG_1380 and MSMEG_6440 may retain partial functionality. Confirmation of the precise mechanistic impact of this novel SV in MTB itself, ideally through gene knockout and complementation, would further solidify its causal role. Second, the mechanism by which the SV-type strains exhibit elevated MICs relative to the empty-vector control remains unresolved. Direct validation, including gene expression quantification and inhibitor assays, is required in future investigation. Third, the truncated proteins should be further investigated in future studies. Fourth, due to the small sample size of our clinical isolate collection, the prevalence and broader clinical significance of this novel *ethA* SV across diverse geographical settings remain to be determined.

In conclusion, this study identifies a novel *ethA* SV, c.135_265del (p.Trp45fs), as a previously unreported cause of ETO resistance, validated by heterologous expression, MIC determination, Western blotting, and AlphaFold 3, and the 16-fold MIC increase conferred by this deletion, together with predicted loss of the C-terminal monooxygenase domain, establishes a compelling genotype–phenotype link. Additionally, our study validates a specific *Rv0678 IS6110* insertion allele, demonstrating the power of long-read sequencing to uncover both known and novel resistance mechanisms. Ultimately, integrating long-read sequencing into routine diagnostic workflows will enhance detection of resistance-associated SVs and guide more effective treatment regimens.

## Data Availability

The datasets presented in this study can be found in online repositories. The names of the repository/repositories and accession number(s) can be found: China National GeneBank Sequence Archive (CNSA), accession no. CNP0008151 (https://db.cngb.org/search/project/CNP0008151/).

## Footnotes

1
https://golgi.sandbox.google.com/
