# Supplementary material for: Long-read sequencing of clinical multidrug-resistant Mycobacterium tuberculosis reveals a novel ethA structural variant conferring ethionamide resistance
Source: Front Microbiol. 2026 Oct 27;17:1929307. doi: 10.3389/fmicb.2026.1929307 (PMC13760529; doi:10.3389/fmicb.2026.1929307)
Supplement: Supplementary file 1 [file Supplementary_file_1.docx]

**Supplementary Figure S1. Hybrid (ONT+Illumina) assembly approach for variant and SV detection**. **(a)** Overlap of variant-containing genes and individual variants identified at the whole-genome scale between the eight MDR and the three susceptible isolates plus H37Rv, using the hybrid assembly approach. Dotted circles denote structural variants (SVs). **(b)** Overlap of SVs identified at the whole-genome scale by BCFtools and SVIM-asm between the MDR and susceptible groups, derived from hybrid assemblies. Dotted circles highlight MDR-specific SVs that were detected by both callers and reside within candidate drug resistance genes of the WHO catalogue. **(c)** Maximum-likelihood phylogenetic tree of the 11 clinical isolates plus H37Rv, based on core single-nucleotide polymorphisms (SNPs) extracted from hybrid (ONT+Illumina) assemblies. **(d)** Stacked bar charts showing the numbers of classified and unclassified mutations found exclusively in the MDR isolates within candidate drug resistance genes of the WHO catalogue, based on hybrid assemblies.

**Supplementary Table S1. Clinical and microbiological characteristics of 11 *Mycobacterium tuberculosis* (MTB) isolates plus the MTB H37Rv reference strain.**

| **Sample** | **Sex** | **Age** | **Xpert MTB/RIF** | **Sputum smear** |
| --- | --- | --- | --- | --- |
| S1 | / | / | / | / |
| S2 | Male | 74 | S | 2+ |
| S3 | Male | 68 | S | 4+ |
| S4 | Male | 66 | S | - |
| R1 | Male | 40 | R | 2+ |
| R2 | Male | 59 | R | 2+ |
| R3 | Female | 62 | R | / |
| R4 | Male | 45 | R | 4+ |
| R5 | Female | 54 | R | / |
| R6 | Male | 85 | R | / |
| R7 | Male | 53 | R | 3+ |
| R8 | Male | 47 | R | - |

S1, the MTB H37Rv reference strain; S2-S4 and R1-R8, 11 clinical isolates; S, rifampicin-susceptible; R, rifampicin-resistant; numerical grade indicating acid-fast bacilli per field of sputum smear microscopy; -, negative; /, not available.

**Supplementary Table S2. Summary statistics of raw long-read sequencing data from the ONT platform.**

| **Sample** | **Num_seqs** | **Sum_len** | **Min_len** | **Avg_len** | **Max_len** | **N50** |
| --- | --- | --- | --- | --- | --- | --- |
| S1 | 467298 | 2660542326 | 1600 | 5693.5 | 94987 | 7884 |
| S2 | 640590 | 3286351160 | 1600 | 5130.2 | 693295 | 6953 |
| S3 | 457031 | 2679283777 | 1600 | 5862.4 | 657713 | 8712 |
| S4 | 478527 | 2802578600 | 1600 | 5856.7 | 470481 | 8626 |
| R1 | 435076 | 2631274065 | 1600 | 6047.8 | 462402 | 9177 |
| R2 | 479754 | 3057988744 | 1600 | 6374.1 | 319812 | 9800 |
| R3 | 553571 | 3168094010 | 1600 | 5723 | 123202 | 8335 |
| R4 | 568998 | 3237467484 | 1600 | 5689.8 | 167918 | 8280 |
| R5 | 196757 | 1743298481 | 1600 | 8860.2 | 174548 | 14361 |
| R6 | 200947 | 1786002922 | 1600 | 8887.9 | 181167 | 14579 |
| R7 | 185067 | 1654111027 | 1600 | 8937.9 | 152883 | 14453 |
| R8 | 169040 | 1615483973 | 1600 | 9556.8 | 144764 | 15433 |

Num_seqs, number of sequences; Sum_len, total summed length; Min_len, minimum read length; Avg_len, average read length; Max_len, maximum read length; N50 statistic, weighted median of the lengths of sequences.

**Supplementary Table S3. Summary statistics of short-read sequencing data from the Illumina platform.**

| **Sample** | **Raw reads** | **Raw bases** | **Clean reads** | **Clean bases** | **Q20_rate** | **Q30_rate** | **GC_content** |
| --- | --- | --- | --- | --- | --- | --- | --- |
| S1 | 6083464 | 912519600 | 5921858 | 873930508 | 97.01% | 91.78% | 65.07% |
| S2 | 6434186 | 965127900 | 6268254 | 924833896 | 97.02% | 91.71% | 65.06% |
| S3 | 12793724 | 1919058600 | 12675184 | 1875618878 | 97.85% | 93.27% | 65.07% |
| S4 | 11677812 | 1751671800 | 11531542 | 1694380330 | 97.77% | 93.29% | 65.09% |
| R1 | 13231818 | 1984772700 | 13093630 | 1935483456 | 97.78% | 93.16% | 65.21% |
| R2 | 10321872 | 1548280800 | 10129284 | 1492567650 | 97.41% | 92.51% | 65.17% |
| R3 | 8551766 | 1282764900 | 8322174 | 1221397638 | 96.98% | 91.61% | 65.03% |
| R4 | 6528590 | 979288500 | 6359920 | 931312844 | 97.05% | 91.90% | 65.14% |
| R5 | 9180692 | 1377103800 | 8934672 | 1308290912 | 97.02% | 91.77% | 64.81% |
| R6 | 7687616 | 1153142400 | 7477166 | 1103437070 | 96.98% | 91.65% | 65.12% |
| R7 | 8539138 | 1280870700 | 8305960 | 1223133506 | 96.98% | 91.65% | 65.11% |
| R8 | 6121634 | 918245100 | 5990514 | 879281748 | 97.25% | 92.28% | 65.07% |

Raw reads, number of reads before data preprocessing; Raw bases, total bases before data preprocessing; Clean reads, number of reads after data preprocessing; Clean bases, total bases after data preprocessing; Q20 rate, percentage of bases with Phred quality score >20; Q30 rate, percentage of bases with Phred quality score >30; GC content, proportion of guanine and cytosine in sequence.

**Supplementary Table S4. Comparative quality metrics of *de novo* genome assemblies generated by different strategies.**

| **Parameter** | **ONT** | **Illumina** | **ONT+Illumina** |
| --- | --- | --- | --- |
| Genome fraction (%) | 99.14 (±0.32) | 97.24 (±0.79) | 99.14 (±0.30) |
| Mismatches per 100kbp | 38.84 (±11.60) | 32.73 (±10.08) | 38.85 (±11.61) |
| Indels per 100 kbp | 7.89 (±2.20) | 5.52 (±1.79) | 8.17 (±2.13) |
| Contigs | 2.17 (±2.33) | 132.50 (±33.52) | 1.00 (±0.00) |

Values represent the mean across all 12 isolates, with standard deviation in parentheses.

**Supplementary Table S5. Assembly statistics for genomes generated from ONT long-read data.**

| **Sample** | **Total length (bp)** | **%GC** | **Contigs** | **Circle** |
| --- | --- | --- | --- | --- |
| S1 | 4423373 | 65.61 | 1 | True |
| S2 | 4437062 | 65.55 | 3 | False |
| S3 | 4419026 | 65.59 | 1 | True |
| S4 | 4561659 | 64.80 | 7 | False |
| R1 | 4411838 | 65.61 | 1 | True |
| R2 | 4413350 | 65.61 | 1 | True |
| R3 | 4399613 | 65.62 | 1 | True |
| R4 | 4420319 | 65.61 | 1 | True |
| R5 | 4415902 | 65.61 | 1 | True |
| R6 | 4473223 | 65.61 | 1 | False |
| R7 | 4518071 | 65.29 | 7 | False |
| R8 | 4423996 | 65.60 | 1 | True |

**Supplementary Table S6. Assembly statistics for genomes generated from Illumina short-read data.**

| **Sample** | **Total length (bp)** | **%GC** | **Contigs** | **Circle** |
| --- | --- | --- | --- | --- |
| S1 | 4335345 | 65.56 | 123 | False |
| S2 | 4349051 | 65.55 | 148 | False |
| S3 | 4336072 | 65.59 | 99 | False |
| S4 | 4299244 | 65.60 | 94 | False |
| R1 | 4323709 | 65.60 | 93 | False |
| R2 | 4329923 | 65.60 | 90 | False |
| R3 | 4309974 | 65.58 | 159 | False |
| R4 | 4316380 | 65.57 | 174 | False |
| R5 | 4319523 | 65.56 | 179 | False |
| R6 | 4352303 | 65.60 | 117 | False |
| R7 | 4225191 | 65.59 | 161 | False |
| R8 | 4286028 | 65.57 | 153 | False |

**Supplementary Table S7. Assembly statistics for genomes generated from the hybrid (ONT+Illumina) assembly data.**

| **Sample** | **Total length (bp)** | **%GC** | **Contigs** | **Circal** |
| --- | --- | --- | --- | --- |
| S1 | 4,425,029 | 65.61 | 1 | True |
| S2 | 4,429,928 | 65.61 | 1 | True |
| S3 | 4,419,960 | 65.61 | 1 | True |
| S4 | 4,420,983 | 65.61 | 1 | True |
| R1 | 4,412,812 | 65.61 | 1 | True |
| R2 | 4,414,756 | 65.61 | 1 | True |
| R3 | 4,401,155 | 65.62 | 1 | True |
| R4 | 4,422,091 | 65.61 | 1 | True |
| R5 | 4,417,463 | 65.60 | 1 | True |
| R6 | 4,472,604 | 65.62 | 1 | True |
| R7 | 4,414,254 | 65.61 | 1 | True |
| R8 | 4,424,599 | 65.60 | 1 | True |

**Supplementary Table S8. List of anti-tuberculosis drugs and their associated 65 candidate resistance genes from the WHO catalogue (2023).**

| **Drug** | **Abbreviation** | **Gene** |
| --- | --- | --- |
| Amikacin | AMK | *bacA*, *ccsA*, *eis*, *rrs*, *Rv2477c*, *whiB6*, *wjiB7* |
| Bedaquiline | BDQ | *atpE*, *IpqB*, *mmpL5*, *mmpS5*, *mtrA*, *mtrB*, *pepQ*, *Rv0678*, *Rv1979c* |
| Capreomycin | CPR | *bacA*, *ccsA*, *rrl*, *rrs*, *Rv2680*, *Rv2681*, *tlyA*, *whiB6* |
| Clofazimine | CFZ | *fbiA*, *fbiB*, *fbiC*, *fgd1*, *mmpL5*, *mmpS5*, *pepQ*, *Rv0678*, *RV1979c*, *Rv2983* |
| Delamanid | DMD | *ddn*, *fbiA*, *fbiB*, *fbiC*, *fgd1*, *ndh*, *Rv2983* |
| Ethambutol | EMB | *aftB*, *embA*, *embB*, *embC*, *embR*, *glpK*, *Rv2477c*, *Rv2752c*, *ubiA* |
| Ethionamide | ETO | *ethA*, *ethR*, *inhA*, *mshA*, *ndh*, *Rv0565c*, *Rv3083* |
| Isoniazid | INH | *ahpC*, *dnaA*, *glpK*, *hadA*, *inhA*, *katG*, *mshA*, *ndh*, *Rv0010c*, *Rv1129c*, *Rv1258c*, *Rv2752c* |
| Kanamycin | KAN | *bacA*, *ccsA*, *eis*, *rrs*, *Rv2477c*, *whiB6*, *whiB7* |
| Levofloxacin | LFX | *glpK*, *gyrA*, *gyrB*, *Rv1129c*, *Rv2477c*, *Rv2752c* |
| Linezolid | LZD | *rplC*, *tsnR*, *rrl* |
| Moxifloxacin | MFX | *glpK*, *gyrA*, *gyrB*, *Rv1129c*, *Rv2477c*, *Rv2752c* |
| Pyrazinamide | PZA | *clpC1*, *panD*, *pncA*, *PPE35*, *rpsA*, *Rv1258c*, *Rv3236c*, *sigE* |
| Rifampicin | RIF | *glpK*, *lpqB*, *mtrA*, *mtrB*, *nusG*, *rpoA*, *rpoB*, *rpoC*, *Rv1129c*, *Rv2477c*, *Rv2752c* |
| Streptomycin | SM | *bacA*, *gid*, *glpK*, *rpsL*, *rrs*, *Rv1258c*, *Rv2477c*, *whiB7* |

**Supplementary Table S9. Mutations identified using ONT assembly within the candidate drug-resistance genes of WHO catalogue**

| **Classification**  **type** | **Variant**  **counts** | **Confidence** | **Mutations** |
| --- | --- | --- | --- |
| Classified  in the WHO  catalogue | 65 | Associated with  resistance | *gyrA* p.Ala90Val, *gyrA* p.Ser91Pro, *gyrA* p.Asp94Asn, *gyrA* p.Asp94Tyr, *gyrA* p.Asp94Ala, *gyrA* p.Asp94Gly, *rpoB* p.Leu430Pro, *rpoB* p.His445Asp, *rpoB* p.Ser450Leu, *Rv0678* c.139dupG (p.Asp47fs), *rpsL* p.Lys43Arg, *rpsL* p.Lys88Arg, *rplC* p.Cys154Arg, *inhA* c.777C>T, *katG* p.Ser315Thr, *pncA* p.Asp12Gly, *pncA* p.Gln10Pro, *pncA* c.12T>C, eis c.10G>A, *embB* p.Met306Val, *embB* p.Met306Ile, *embB* p.Met306Ile, *embB* p.Gly406Asp, *rrs* n.1401A>G |
|  |  | Associated with  resistance-interim | *pncA* c.528delC (p.Thr177fs)*, pncA* p.Ala46Val*, ethA* c.1114delA (p.Met372fs) |
|  |  | Uncertain  significance | *dnaA* p.Gly231Arg, *dnaA* p.Ser261Thr, *dnaA* p.Ser363Arg, *gyrB* p.Thr500Ala, *Rv0565c* p.Gly335Glu, *rpoB* p.Ile491Leu, *rpoB* p.Glu807Ala, *rpoC* p.Gly332Arg, *rpoC* p.Lys445Arg, *rpoC* p.Val483Gly, *rpoC* p.Asp747Ala, *rpoC* p.Pro1040Arg, *rpoC* p.Arg1083Gly, *Rv1129c* p.Gly446Val, *Rv1129c* p.Val337Ala, *Rv1129c* c.22A>G, *inhA* p.Ala124Val, *katG* p.Val450Ala, *katG* p.Met176Thr, *katG* p.Trp91Arg, *pncA* p.Val180Leu, *ahpC* c.81C>T, *ahpC* c.51G>A, *embA* c.16C>G, *embA* p.Pro2Thr, *embA* p.Gln497Arg, *embB* p.Ile563Leu, *ethA* p.Phe431Leu, *ethA* p.Ser266Arg, *ethA* p.Trp116Cys, *ethA* p.Ser15Pro, *whiB6* p.Ala77Val, *gid* p.Leu101Pro |
|  |  | Not associated with resistance-interim | *fgd1* c.504G>C, *rpoC* c.579G>A, *rpoC* c.2922G>A, *gid* c.291C>G |
|  |  | Not associated with resistance | *glpK* p.Gly191dup |
| Unclassified  in the WHO  catalogue | 23 | / | *gyrA* p.Ile287Thr, *Rv0565c* p.Phe225Ser, *Rv0565c* p.Met208Ile, *Rv0565c* p.Tyr146_Tyr152del, *hadA* p.His74Tyr, *mmpL5* p.Gln665His, *Rv0678* c.59_60ins1358bp (p.Gln22fs), *Rv1129c* p.Ala141Gly, *katG* p.Phe408Val, *katG* c.12G>A, *PPE35* p.Asp147Ala, *Rv1979c* c.2204A>T, *pncA* c.417_424dupGCGCCAGA (p.Thr142fs), *Rv2752c* p.Lys115_Cys116insTer, *Rv2752c* c.1756C>T, *Rv2752c* c.1770C>T, *Rv2752c* c.4660A>G, *whiB7* c.54T>C, *mtrA* p.Glu150Gly, *clpC1* p.Met201Ile, *embB* p.Gly443Asp, *ethA* c.1006_1007dupGA, *ethA* c.135_265del (p.Trp45fs) |

**Supplementary Table S10. Mutations identified using hybrid (ONT+Illumina) assembly within the candidate drug-resistance genes of WHO catalogue**

| **Classification**  **type** | **Variant**  **counts** | **Confidence** | **Mutations** |
| --- | --- | --- | --- |
| Classified  in the WHO  catalogue | 65 | Associated with  resistance | *gyrA* p.Ala90Val, *gyrA* p.Ser91Pro, *gyrA* p.Asp94Asn, *gyrA* p.Asp94Tyr, *gyrA* p.Asp94Ala, *gyrA* p.Asp94Gly, *rpoB* p.Leu430Pro, *rpoB* p.His445Asp, *rpoB* p.Ser450Leu, *Rv0678* c.139dupG (p.Asp47fs), *rpsL* p.Lys43Arg, *rpsL* p.Lys88Arg, *rplC* p.Cys154Arg, *inhA* c.777C>T, *katG* p.Ser315Thr, *pncA* p.Asp12Gly, *pncA* p.Gln10Pro, *pncA* c.12T>C, eis c.10G>A, *embB* p.Met306Val, *embB* p.Met306Ile, *embB* p.Met306Ile, *embB* p.Gly406Asp, *rrs* n.1401A>G |
|  |  | Associated with  resistance-interim | *pncA* c.528delC (p.Thr177fs)*, pncA* p.Ala46Val*, ethA* c.1114delA (p.Met372fs) |
|  |  | Uncertain  significance | *dnaA* p.Gly231Arg, *dnaA* p.Ser261Thr, *dnaA* p.Ser363Arg, *gyrB* p.Thr500Ala, *Rv0565c* p.Gly335Glu, *rpoB* p.Ile491Leu, *rpoB* p.Glu807Ala, *rpoC* p.Gly332Arg, *rpoC* p.Lys445Arg, *rpoC* p.Val483Gly, *rpoC* p.Asp747Ala, *rpoC* p.Pro1040Arg, *rpoC* p.Arg1083Gly, *Rv1129c* p.Gly446Val, *Rv1129c* p.Val337Ala, *Rv1129c* c.22A>G, *inhA* p.Ala124Val, *katG* p.Val450Ala, *katG* p.Met176Thr, *katG* p.Trp91Arg, *pncA* p.Val180Leu, *ahpC* c.81C>T, *ahpC* c.51G>A, *embA* c.16C>G, *embA* p.Pro2Thr, *embA* p.Gln497Arg, *embB* p.Ile563Leu, *ethA* p.Phe431Leu, *ethA* p.Ser266Arg, *ethA* p.Trp116Cys, *ethA* p.Ser15Pro, *whiB6* p.Ala77Val, *gid* p.Leu101Pro |
|  |  | Not associated with resistance-interim | *fgd1 c.504G>C, rpoC c.579G>A, rpoC c.2922G>A, gid c.291C>G* |
|  |  | Not associated with resistance | *glpK* p.Gly191dup |
| Unclassified  in the WHO  catalogue | 24 | / | *gyrA* p.Ile287Thr, *Rv0565c* p.Phe225Ser, *Rv0565c* p.Met208Ile, *Rv0565c* p.Tyr146_Tyr152del, *hadA* p.His74Tyr, *mmpL5* p.Gln665His, *Rv0678* c.59_60ins1358bp (p.Gln22fs), *Rv1129c* p.Ala141Gly, *katG* p.Phe408Val, *katG* c.12G>A, *PPE35* p.Asp147Ala, *Rv1979c* c.2204A>T, *pncA* c.417_424dupGCGCCAGA (p.Thr142fs), *Rv2752c* p.Lys115_Cys116insTer, *Rv2752c* c.1756C>T, *Rv2752c* c.1770C>T, *Rv2752c* c.4660A>G, *whiB7* c.54T>C, *mtrA* p.Glu150Gly, *clpC1* p.Met201Ile, *embB* p.Gly443Asp, *ethA* c.1006_1007dupGA, *ethA* c.135_265del (p.Trp45fs), *rpoB* c.188_252dupGCTGGCGCGAATCCGCCGCCGAGCGGGGTGATGTCAACCCAGTGGGTGGCCTGGAAGAGGTGCTC (p.Tyr85fs) |

**Supplementary Table S11. Distribution of the six representative mutations across the 11 clinical *Mycobacterium tuberculosis* (MTB) isolates plus the H37Rv reference strain.**

| Sample | Lineage | *pncA* c.528delC | *Rv0678* c.139dupG | *ethA* c.1114delA | *pncA* c.417_424dupGCGCCAGA | *Rv0678* c.59_60ins1358bp | *ethA* c.135_265del |
| --- | --- | --- | --- | --- | --- | --- | --- |
| S1 (H37Rv) | 4 | – | – | – | – | – | – |
| S2 | 4 | – | – | – | – | – | – |
| S3 | 2 | – | – | – | – | – | – |
| S4 | 2 | – | – | – | – | – | – |
| R1 | 2 | – | – | – | – | – | – |
| R2 | 2 | – | – | – | – | – | – |
| R3 | 2 | – | – | – | + | – | – |
| R4 | 2 | – | – | – | – | – | – |
| R5 | 2 | – | – | – | – | – | – |
| R6 | 4 | – | – | – | – | – | + |
| R7 | 2 | – | + | – | – | – | – |
| R8 | 2 | + | – | + | – | + | – |

+, mutation present; –, mutation absent. The six mutations comprise three previously classified variants (*pncA* c.528delC, *Rv0678* c.139dupG, *ethA* c.1114delA) and three unclassified variants (*pncA* c.417_424dupGCGCCAGA, *Rv0678* c.59_60ins1358bp, *ethA* c.135_265del).
